# Diet-Nutrition Information Seeking, Source Trustworthiness, and Eating Behavior Changes: An International Web-Based Survey

**DOI:** 10.3390/nu15214515

**Published:** 2023-10-25

**Authors:** Maria A. Ruani, Michael J. Reiss, Anastasia Z. Kalea

**Affiliations:** 1 Curriculum, Pedagogy and Assessment, Institute of Education, Faculty of Education and Society, University College London, London WC1H 0AL, UK; m.reiss@ucl.ac.uk; 2 The Health Sciences Academy, London SW6 5UA, UK; 3 Faculty of Medical Sciences, Division of Medicine, University College London, London WC1E 6BT, UK; a.kalea@ucl.ac.uk; 4 Institute of Cardiovascular Science, University College London, London WC1E 6DD, UK

**Keywords:** nutrition misinformation, literacy, eating behavior change, dietary change, sources of information, information-seeking behaviors, source trustworthiness, public health, communication, social media, misinformed beliefs

## Abstract

To understand the extent to which different sources of diet and nutrition information are sought, trusted, and relied upon for making dietary changes, the present international web-based survey study gauged participants’ (*n* = 3419) diet-nutrition information-seeking behaviors from 22 interpersonal and general sources with varying quality, trust levels in these sources, and reliance on each source for making dietary changes. Qualitative insights were also captured regarding trustworthiness formation. The results revealed a disconnect between source popularity and perceived trustworthiness. While nutrition–health websites, Google–Internet searches, and diet–health books were most commonly consulted, participants placed the highest level of trust in nutrition scientists, nutrition professionals, and scientific journals, suggesting that frequent information seeking from a subpar source may not be a reliable predictor of the level of trust assigned to it. Although the frequency of source-seeking behaviors and source trustworthiness both contributed to dietary changes, the latter appeared to have a more pronounced influence. When a source was less trusted, there was a reduced likelihood of relying on it for changing diet. Additionally, source seeking may not always translate into effective dietary change, as shown by the less strong correlation between the two. These associations significantly differed depending on the source.

## 1. Introduction

The proliferation of diet-nutrition information sources in today’s digital era has brought both opportunities and challenges. While access to information has increased, so has the prevalence of misinformation and misleading claims regarding diet and nutrition [1,2]. Understanding how individuals navigate this landscape of information and evaluate the trustworthiness of sources is crucial for combating the risks associated with misinformation and potentially ill-informed dietary decisions with harmful health consequences [1].

Widespread misinformation, disinformation, and malinformation [3] related to diet and nutrition have been fueled by various factors, including the ease of disseminating information through digital platforms and the vast array of sources available to individuals [4,5,6,7]. This abundance of information, however, comes with inherent challenges, as the accuracy and reliability of the information are not guaranteed [8,9]. Misinformation in diet and nutrition can encompass a wide range of deceptive claims, unfounded recommendations, and pseudoscientific theories, often driven by commercial or political interests or personal beliefs [1]. This can mislead individuals, undermine trust in science, and contribute to the adoption of potentially harmful dietary behaviors [10,11]. The consequences of misinformation in this domain extend beyond individual health outcomes, impacting public health, healthcare systems, and societal well-being [5,12,13,14,15]. Recognizing the prevalence and problems associated with misinformation in diet and nutrition is crucial for fostering informed decision making, promoting evidence-based practices, and addressing the risks and implications of false information and wrongful advice on public health [2].

Various infodemics reviews have examined the channels through which health misinformation spreads, highlighting social media platforms, influential figures, and mainstream media as major contributors [5,6]. However, the extent to which individuals actively seek diet-nutrition information from these sources in comparison to others remains less elucidated. Furthermore, the digital realm is dynamic and ever-evolving [14], continuously introducing new venues for disseminating diet and nutrition information. This adds complexity to the study of diet-nutrition misinformation and stresses the need for in-depth examination.

Gaining a comprehensive understanding of individuals’ abilities to discern between low- and high-quality sources is of the utmost importance. Currently, there is limited knowledge about how effectively individuals can distinguish reliable from misinforming sources in diet and nutrition. Assessing the reliability of sources and determining the accuracy of information proves to be an arduous task, even for trained researchers and nutrition professionals [4]. Hence, the investigation of source quality discernment, as a sub-category of information literacy [16,17,18], opens up possibilities for developing interventions that actively deter individuals from relying on sources of inferior quality when seeking guidance on diet or nutritional advice.

The soundness of diet-nutrition information trustworthiness evaluations, and whether these evaluations approximate source quality, are not yet understood. The significance of trustworthiness judgments in fighting misinformation can be viewed from two perspectives. Firstly, by promoting trust in science, scientific institutions, and credentialed professionals, individuals may be more likely to rely on evidence-based information, reducing their vulnerability to misinformation [1,19,20,21,22] while likely increasing the odds of better informed decisions regarding diet and nutrition. However, the relationship between trust levels and the true quality of diet-nutrition information sources sought remains an area that lacks exploration. Secondly, it is equally important to address the issue of trust in inferior quality sources like social media platforms (e.g., Facebook, WhatsApp forwarded messages), popular personalities, and mainstream media (e.g., magazines, television, newspapers), which are often riddled with misinformation [5,6]. By deepening our insight about the sources that individuals seek and trust to inform their dietary choices, including whether trustworthiness judgements align with information source quality, we can design tailored educational initiatives to mitigate the risk of misinformation and encourage safer dietary change decisions.

Additionally, while there are available empirical data looking at perceived utility and trustworthiness in the context of health-information-seeking behaviors [23], the informational predictors of eating behavior changes remain unclear. Only one study has looked at information sources of varying quality used for dietary change, where high reliance on sources such as social media, WhatsApp messages, and famous personalities for making changes in eating behavior was associated with holding more nutrition–health misinformation [1]. Greater susceptibility to misinformation when individuals rely on poorer quality sources of diet-nutrition information for making dietary changes is an area of great concern, as this may in turn increase the risk of health harm.

However, no previous research has specifically addressed the question of how diet-nutrition information source seeking and the level of trust assigned to these sources relate to the reliance on them for making dietary changes. Therefore, to understand the extent to which different sources of diet and nutrition information are sought, trusted, and relied upon for making dietary changes, a web-based survey study was designed and distributed internationally to assess the respondents’ diet-nutrition information source-seeking behaviors, the level of trust attributed to each of the 22 interpersonal and general sources, and their influence on the frequency of changes in eating behavior. On this premise, the present survey study attempts to explore the following:

- The respondents’ self-reported diet-nutrition information-seeking frequency per source (i.e., diet-nutrition information source-seeking behaviors).
- The degree of trust that respondents placed in various sources of nutrition information or dietary advice (i.e., diet-nutrition information source trustworthiness), alongside their own interpretation of trustworthy diet-nutrition information or advice.
- The self-reported influence of different sources of diet and nutrition information or advice on the frequency of changes in food and eating behaviors (i.e., diet-nutrition information source reliance for making dietary changes).
- The relationships between diet-nutrition information-seeking frequencies from different sources, the trust levels in these sources, and the extent of self-reported dietary changes made based on their information (i.e., potential correlations between source-seeking behaviors, trustworthiness, and reliance for making dietary changes).

## 2. Materials and Methods

### 2.1. Study Design and Population

An anonymous online survey questionnaire was formulated to gauge respondents’ diet-nutrition information source-seeking frequencies and trust levels in different sources, as well as the influence of these on changes in eating behavior. The adoption of a non-probabilistic, opportunity sampling method enabled a convenient distribution of the survey amongst some 100,000 email subscribers and students of The Health Sciences Academy, who represent a population [24] with an above-average education and an interest in nutritional matters predominantly from the United Kingdom, the United States, and Australia (among circa 170 other countries), and the subsequent gathering of a relatively large number of online voluntary responses between 6 June and 6 October 2019.

A total of 3487 respondents participated in the survey. Subsequently, 44 minors (respondents aged 17 or below) were excluded from the analysis. Among the remaining 3443 participating adults, 3419 provided informed consent for their anonymized responses to be included in the analysis. Consequently, the final sample for this study comprised 3419 respondents.

### 2.2. Ethical Considerations

Ethical approval for this study was granted by the Research Ethics Committee at University College London, under the reference number Z6364106/2018/06/67. The survey design, distribution, and data processing were in accordance with the approval guidelines.

Participation in the survey was voluntary and presented no known risks or direct benefits, with no associated compensation or financial incentives. Informed consent was requested on the survey’s front page before granting access to the rest of the questionnaire. Responses were collected through a web-based form on the SurveyMonkey platform, with participants given the option to skip questions or exit the questionnaire at any point without repercussions. To prevent duplicate responses, survey settings were configured to restrict multiple submissions from a single participant. Additionally, responses were collected without requiring formal submission of the form. The survey was administered via a web link included in the invitation email and could be completed on any device with a browser and Internet access.

### 2.3. Instrument Measures and Outcomes

The survey instrument developed for this research study was a questionnaire in English, consisting of a voluntary consent question on the front page, followed by 16 questions (several with sub-questions) in two main parts, as described below.

#### 2.3.1. General Demographics and Characteristics

This first part collected participant data on country of residence, employment status, age, gender, number of physical and digital books owned, popular diets tried in the past, current dietary pattern, self-reported health status, body weight categorization, physical activity levels, extent of interest in nutrition and health, and nutrition profession involvement or lack thereof.

Supplementary Table S1 presents the sociodemographic characteristics of the respondents, exhibiting a majority of female participants (78.2%). This is likely attributable to the prevailing demographics of The Health Sciences Academy as well as the established trend of females being considerably more likely to engage in fully voluntary online surveys, particularly those pertaining to nutrition and health [25,26,27]. A total of 137 countries worldwide were represented, with 35.9% of respondents being from the United Kingdom, 15.4% from the United States, 6.5% from Canada, 5.6% from Australia, 5.6% from India, 2.2% from Ireland, and 28.8% from another 131 countries. Adults aged 18 to 70 or older were included in the sample, with the majority being employed (69.1%) and ages 21 to 65 being uniformly represented (92.7%). Over a third of respondents reported owning 100 to 499 books (34.7%) and 16.5% of them reported owning more than 500 books [28]. When asked about their current diet, the majority stated that they consume plant foods (99.7%) in different proportions, but most commonly a balance of plant foods and animal foods (50%). The most popular diets tried in the past were ‘low-carb’ (44.2%), ‘low-calorie’ (41.7%), ‘low-fat’ (38%), ‘intermittent fasting’ (37.2%), ‘vegetarian’ (36.8%), and ‘high-protein’ (35.3%), although dietary overlap was possible. The respondents’ self-reported health was mostly good or better (48.8% ‘good’ and 24.2% ‘great’), their self-reported weight categorization was largely in the normal (52.8%) and slightly overweight (22.6%) ranges, and their physical activity levels were primarily in the active groups (‘moderately active’ 38.8%, ‘active’ 35.2%, and ‘very active’ 16.3%). Respondents were also asked about their interest in nutrition and health and nearly all (99.5%) expressed being interested (62.7% ‘extremely interested’, 28.5% ‘very interested’, and 8.4% ‘interested’). Lastly, although 58.9% had no involvement in the nutrition profession, 17.9% affirmed being a nutrition professional, and 23.2% said that they were studying for this. Additional participants’ characteristics can be found in Supplementary Table S1.

#### 2.3.2. Diet-Nutrition Information Seeking, Source Trustworthiness, and Eating Behavior Changes

The second part of the survey was designed to measure respondents’ diet and nutrition information-seeking behaviors, assigned source trustworthiness, and self-reported frequency of changes in food and eating behaviors based on nutrition information or dietary advice from 22 possible sources (Supplementary Table S2).

Eleven of these sources were interpersonal (individuals such as one’s medical doctor or GP, nutrition professionals, influencers followed on social media, family members, friends, colleagues, or peers). The other 11 were general sources of nutrition information or dietary advice (such as Google or Internet searches, diet or health books, nutrition or health websites, social media, and scientific journals or academic manuals).

Possible sources were listed in a randomly rotated order for each participant within each matrix-style question, asking the following:

- “How often do you seek nutrition information/dietary advice from the following individuals/sources?”
- “How much do you trust the nutrition information/dietary advice given by the following individuals/sources?”
- “Have you changed the way you eat or what you eat based on nutrition information/dietary advice from the following individuals/sources?”

The frequency of diet and nutrition information-seeking behaviors and the frequency of eating behavior changes for each of the listed sources of nutrition information or dietary advice were assessed using a scale comprising ‘never’, ‘rarely’, ‘sometimes’, ‘often’, and ‘always’ (information seeking) or ‘all the time’ (eating changes). The trustworthiness attributed to each source was evaluated using a scale of ‘least trustworthy’, ‘not very trustworthy’, ‘trustworthy’, ‘very trustworthy’, and ‘most trustworthy’.

In addition, the survey gathered qualitative data using an open-ended question to investigate the participants’ own interpretation of trustworthiness of diet-nutrition information or advice. The specific question asked was “In your own words, what does ‘trustworthy’ nutrition information/dietary advice mean to you?”

### 2.4. Statistical Analysis

The statistical analysis of the results was conducted using Microsoft^®^ Excel^®^ for Microsoft 365 MSO (Version 2202, Build 16.0.14931.20118, 32-bit) and SurveyMonkey’s statistical analysis features.

Descriptive statistics were applied for the analysis of the collected data, such as computing numbers of responses (# observations), percentages, and mean values. For cross-tabulations comparing survey answer choices among different groups of respondents, *p* values and weighted averages of aggregated data were obtained. A *p*-value below 5% (*p* < 0.05) was deemed to be statistically significant. To provide a ranking of the data related to source information-seeking and dietary change frequencies, responses were weighted as follows: ‘never’, 0; ‘rarely’, 1; ‘sometimes’, 2; ‘often’, 3; and ‘always’ or ‘all the time’, 4. For the same purpose, source trustworthiness responses were weighted as follows: ‘least trustworthy’, 0; ‘not very trustworthy’, 1; ‘trustworthy’, 2; ‘very trustworthy’, 3; and ‘most trustworthy’, 4. The weighted averages were then calculated and sorted in descending order.

Spearman correlations (*r_s_*) were employed to assess the associations between specific variables. *r_s_* values ranged from −1 to 1, where negative values indicated inverse associations between the variables. The following reference absolute values were considered: *r_s_* = 0—no correlation, *r_s_* between 0.0 and 0.2—very weak correlation, *r_s_* between 0.2 and 0.4—weak correlation, *r_s_* between 0.4 and 0.6—moderate correlation, *r_s_* between 0.6 and 0.8—strong correlation, *r_s_* between 0.8 and 1.0—very strong correlation, and *r_s_* = 1—perfect correlation [29].

Within the context of our study, large numbers of cross-tabulations were performed, and the majority of them did not reach statistical significance, as determined through chi-squared testing. For example, no discernible patterns emerged regarding the influence of participant age or country of residence on their responses. As a result, we decided not to present the outcomes of such cross-tabulations in the subsequent sections.

The Smart Coding Tool of the qualitative research software MaxQDA Analytics Pro 2022 (Release 22.5.0) by VERBI Software was employed to distinguish the themes present in the participants’ responses to the open-ended question concerning the trustworthiness of diet-nutrition information or advice, including the quantification of the number of responses that aligned with each identified theme. A primary coder initiated the organization and thematic analysis of the qualitative data, generating initial codes by identifying repeated phrases and words, from which primary coding keywords were extracted and quantified using the aforementioned software. The identified patterns were subsequently consolidated and combined into fourteen distinct overarching themes, with each verified for accurate data representation, including the quantification of the number of responses that aligned with them. Two additional researchers independently reviewed and validated the consistency and accuracy of coding and theme development, assisting in the selection of illustrative examples for each theme.

## 3. Results

### 3.1. Diet-Nutrition Information-Seeking Behaviors

Table 1 presents a ranking of sources of nutrition information or dietary advice, arranged in order of the frequency with which participants reported seeking information from these sources. The three most sought-after sources included nutrition or health websites (mean information-seeking frequency = 2.5, WAVG), Google or Internet searches (2.4), and diet or health books (2.2). Conversely, fiction books or movies (0.38), famous personalities, actors or presenters (0.5), and gym instructors or personal trainers (0.92) were ranked as the least sought-after sources of diet-nutrition information. Social media and influencers followed on social media were ranked in the eighth and fourteenth places, with moderate information-seeking scores (1.35 and 1.22, respectively). Scientific journals or academic manuals were in the fourth place (1.86), closely followed by science news publications (1.85), nutrition scientists and PhDs (1.76), and nutrition professionals (1.76). Diet-nutrition information seeking from one’s own medical doctor or GP (0.99) and from celebrity doctors or experts (0.99) were less common and in the seventeenth and eighteenth places, respectively.

### 3.2. Assigned Source Trustworthiness in Relation to Nutrition Information or Dietary Advice

Table 2 displays the degree of trust that respondents placed in various sources of nutrition information or dietary advice. The sources are ranked based on the level of trust assigned by participants, with the most trusted sources being nutrition scientists and PhDs (mean trust score = 2.84), nutrition professionals (2.70), and scientific journals or academic manuals (2.58), and the least trusted sources comprising fiction books or movies (0.43), famous personalities, actors, or presenters (0.56), and social media (0.79). Greater trust was placed on nutrition information or dietary advice from science news publications (2.33), nutrition or health websites (2.11), and diet or health books (2.0) than from nurses or health coaches (1.89) and one’s own medical doctor or GP (1.73).

#### Qualitative Perspectives on Trustworthiness in Diet-Nutrition Information

Supplementary Table S3 lists fourteen descriptive themes that were identified from 1744 responses freely typed by participants with their interpretation of/viewpoints about trustworthy diet-nutrition information or advice, along with examples of their writing.

Evidence-based information was highly valued, with 44.7% of responses emphasizing the importance of science and research in their trustworthiness evaluations. Including references to research or being able to trace the origin of the information and verify its sources were also crucial, as this was mentioned in 22.7% of responses. Familiar, credible, or reputable sources were focal for 18.6% of respondents, preferring information from peer-reviewed journals or professionals with the necessary credentials. Practicality and applicability were often deemed important, with 17.1% of responses highlighting the need for realistic, feasible, and sensible advice in order to trust it more. Truthful, accurate, and reliable information was valued by 10.7% of respondents, who stressed the need for objective, factual, and transparent/honest advice. Unbiased information was sought by 7.5% of respondents, who were wary of hidden agendas, conflicts of interest, or one-sided perspectives. Established information, tested over a longer period, was important for 7.1% of respondents, who preferred advice based on long-term studies or reproducible results. Peer-reviewed or verified information was valued by 6.5% of respondents, who trusted studies published in academic journals or advice checked by experts. Personalized advice was important for 4.1% of respondents, who deemed tailored information based on individual circumstances as more trustworthy. Comprehensive information, including strengths and limitations, was valued by 3.6% of respondents, who appreciated balanced, well-rounded advice, such as that airing its risks and benefits. Rational and explanatory information was sought by 3% of respondents, who favored logical explanations and educational content. Avoiding fads or miracle solutions was important for 2.9% of respondents, who were skeptical of quick fixes or magic bullets. Large sample sizes and significant findings were instrumental for 2.6% of respondents, who trusted information based on extensive research and robust data. Simple and specific information was valued by 2.2% of respondents, who preferred clear, concise, and comprehensible advice. Further information, including examples of participant responses for each descriptive theme, is available in Supplementary Table S3.

### 3.3. Reliance on Sources of Varying Quality for Making Dietary Changes

Table 3 details a ranking of the most to least relied-upon sources for making dietary changes based on the frequency of those changes reported for each source. The most relied-upon sources for making dietary changes involved nutrition professionals (mean dietary change frequency = 2.13) and nutrition scientists and PhDs (2.08), closely followed by nutrition or health websites (1.98) and diet or health books (1.91). Google or Internet searches (1.53) had a greater influence on eating behavior changes than nurses or health coaches (1.29) and one’s own medical doctor or GP (1.29). The least relied-upon sources for making dietary changes were fiction books or movies (0.29), famous personalities, actors or presenters (0.44), social media (0.77), and influencers followed on social media (0.79).

#### Greater Change Reliance on Lower Quality Diet-Nutrition Information Sources When Owning Fewer Books

Supplementary Table S5 presents a comparison of self-reported dietary change frequencies for each of six subpar sources, depending on the number of physical and digital books owned by participants. It was observed that participants who owned a limited number of books relied more heavily on lower quality sources of nutrition information or dietary advice for changing their eating behaviors, compared to those owning a larger number. For example, among the 91 participants who often relied on social media for making dietary changes, over a quarter (26.4%, *p* < 0.05) owned 9 or fewer books, while a mere 1.1% owned 1000 or more books. This trend was observed across all subpar sources.

### 3.4. Associations between Source Use, Trust, and Reliance for Changing Eating Behavior

#### 3.4.1. Strongest Correlations Found between Source Trustworthiness and Reliance for Making Dietary Changes across All Sources

**Figure 1:**
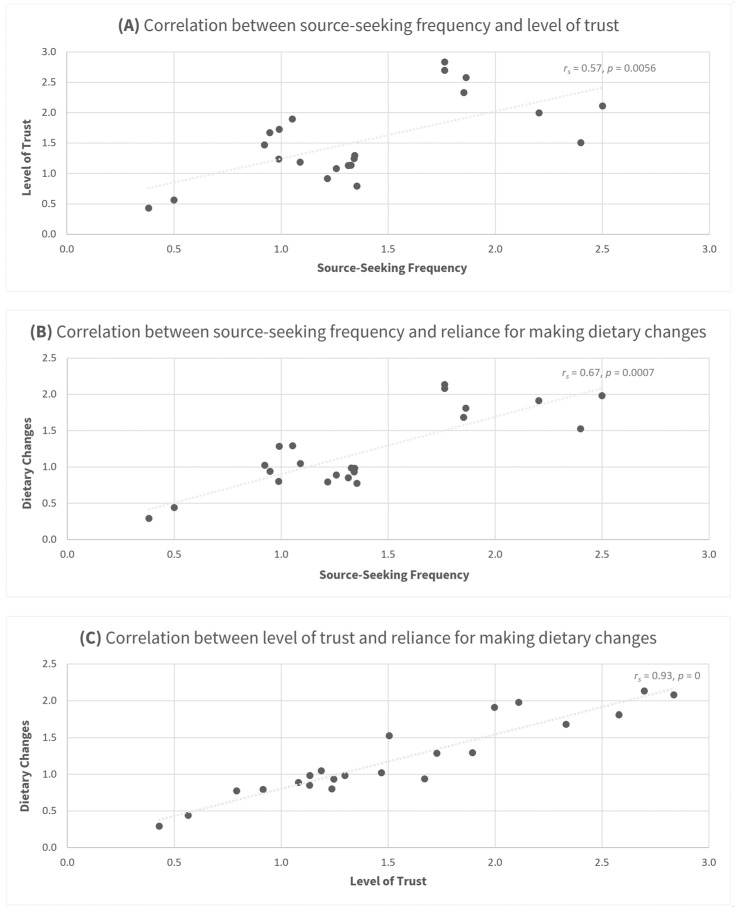
Scatter charts exhibiting correlations between diet-nutrition–information-seeking frequencies from 22 sources, trust levels in these sources, and extent of self-reported dietary changes made based on information from these sources. (**A**) Moderate correlation between source-seeking frequency and level of trust (*r_s_* = 0.57, *p* = 0.0056). (**B**) Strong correlation between source-seeking frequency and reliance for making dietary changes (*r_s_* = 0.67, *p* = 0.0007). (**C**) Very strong correlation between level of trust and reliance for making dietary changes (*r_s_* = 0.93, *p* = 0). Note: WAVG values from Table 1, Table 2 and Table 3 were paired up for each source and subsequently plotted in the relevant scatter chart. For example, the mean trust score of 2.84 for nutrition scientists and PhDs was paired with their mean dietary change frequency of 2.08 and plotted jointly in chart (**C**).

Spearman correlations were utilized to examine the relationship between the frequency of diet-nutrition information seeking from 22 sources, the level of trust in these sources, and the reliance on them for altering eating behavior. As illustrated in Figure 1A, the correlation between source-seeking frequency and level of trust was moderate (*r_s_* = 0.57, *p* = 0.0056). The correlation between source-seeking frequency and reliance for making dietary changes was strong (*r_s_* = 0.67, *p* = 0.0007) (Figure 1B). The correlation between level of trust and reliance for making dietary changes was very strong (*r_s_* = 0.93, *p* = 0) (Figure 1C).

#### 3.4.2. Disparities in Information Seeking, Trustworthiness, and Reliance for Dietary Changes Depending on Source Type

Supplementary Table S4 depicts cross-tabulations of source-seeking frequencies with levels of trust and reliance for making dietary changes. For each source, the assigned levels of trust relative to seeking behavior frequencies differed, and so did the reliance for making dietary changes relative to the source-seeking frequency.

The large majority of nutrition–health website information seekers exhibited greater trust in this source (94.3% in the ‘always’ group, 90.9% in the ‘often’ group, 74.5% in the ‘sometimes’ group) compared to non-seekers and rare-seekers (41.6% in the ‘never’ group and 56.9% in the ‘rarely’ group; *p* < 0.05). Likewise, a greater proportion of nutrition–health website information seekers relied on such information for performing dietary changes (93.6% in the ‘always’ group, 86.2% in the ‘often’ group, and 67.9% in the ‘sometimes’ group) than non-seekers and rare-seekers (24.8% in the ‘never’ group and 41.1% in the ‘rarely’ group; *p* < 0.05). Similar patterns were observed among participants who obtained diet-nutrition information from subpar/lower quality sources such as social media, magazines or newspapers, diet or health books, and Google or Internet searches, but not from higher quality sources such as scientific journals, academic manuals, and science news publications, where non- and rare-seekers also trusted these sources despite using them less or not at all. For example, while the majority of non-seekers and rare-seekers of diet-nutrition information from scientific journals or academic manuals expressed trust in these sources (76.5% in the ‘never’ group and 86% in the ‘rarely’ group), only a smaller proportion of them actually relied on scientific journals or academic manuals for making dietary changes (21.6% in the ‘never’ group and 47.1% in the ‘rarely’ group). These trust percentages represent the sum of ‘most trustworthy’, ‘very trustworthy’, and ’trustworthy’ values depicted in Supplementary Table S4, where additional comparisons across various sources can be found.

## 4. Discussion

The present survey study aimed to investigate the respondents’ diet-nutrition information-seeking behaviors from a selection of 22 interpersonal and general sources, nutrition information, or dietary advice. The level of trust assigned to each source was also evaluated, along with its influence on the frequency of self-reported changes in eating behaviors. Additionally, we examined whether the frequency of source use was correlated with source trustworthiness and with source reliance for making dietary changes.

### 4.1. Source Popularity and Trustworthiness

The most commonly sought-after sources of diet-nutrition information were nutrition or health websites, Google or Internet searches, and diet or health books. Despite these sources being the most frequently consulted, they were not necessarily regarded as the most trustworthy. Instead, participants placed the greatest trust in nutrition scientists and PhDs, nutrition professionals, and scientific journals or academic manuals. These results highlight a dichotomy between the popularity versus the perceived trustworthiness of information sources. The observed preference for easily accessible online sources of information suggests that convenience may be a significant factor driving information-seeking behaviors. However, the high trust scores for scientifically driven sources indicate that credibility remains paramount when it comes to trusting nutrition information and dietary advice. The existing literature points out that individuals commonly seek information from sources that they may not inherently trust [30], and supports the notion that those who place trust in science (e.g., scientists, scientific institutions, healthcare professionals) are less likely to be susceptible to health misinformation [19].

Studies examining susceptibility to health misinformation caution that relying on subpar sources, such as social media, to obtain information does increase the likelihood of falling for health misinformation [19]. In our study, higher frequencies of seeking diet-nutrition information from lower quality sources, such as social media, magazines or newspapers, diet or health books, and Google or Internet searches, were found to be positively correlated with greater assigned trustworthiness of these sources. This suggests that individuals who actively sought information from these subpar sources tended to place a higher level of trust in them compared to non-seekers and rare-seekers, potentially making them more susceptible to diet-nutrition misinformation. On the other hand, participants who did not actively seek information from scholarly materials like scientific journals or academic manuals still expressed trust in them, even when they were less likely to utilize these resources or did not use them at all, indicating that trust in higher quality sources is influenced by factors other than seeking behaviors.

Qualitatively, respondents deemed diet-nutrition information as trustworthy when it was evidence-based, with references to research or verifiable sources, and came from familiar, credible, or reputable sources, such as professionals with the necessary credentials. Practicality, applicability, accuracy, and reliability were also important factors, as many participants sought realistic and feasible advice that was truthful, objective, and transparent. Unbiased information, free from commercial interests, tested over time, and peer-reviewed or verified, was highly valued, along with personalized advice that took individual circumstances into account. Rational explanations, the avoidance of fads or miracle solutions, and statistically significant findings based on large samples also contributed to the trustworthiness evaluations. A small proportion of respondents preferred clear, concise, and understandable advice that combined these attributes, valuing information that was simple and specific. These factors should be further explored in future investigations on the formation of trustworthiness judgements [31], integrated into the design or refinement of information trustworthiness evaluation frameworks [32,33], and utilized in shaping interventions to mitigate diet-nutrition misinformation susceptibility risk [19], including inoculation (pre-bunking) and correction (debunking) strategies [34,35].

### 4.2. Source Influence on Eating Behavior Changes

Our analysis indicates that participants relied on certain types of sources more than others for making dietary changes, with higher quality sources having a greater influence on modifying eating behavior. Encouragingly, lower quality sources resulted in fewer self-reported dietary changes. This coincides with a prior study by Ruani and Reiss which also found a lesser influence of subpar sources on eating behavior changes during the early stages of the COVID-19 pandemic [1].

Although famous personalities, actors, presenters, and influencers followed on social media enjoy widespread popularity, our results show that these sources were amongst the least relied upon for making dietary changes, along with fiction books or movies and social media. Instead, participants most heavily relied on nutrition professionals and nutrition scientists or PhDs for changing eating behaviors, followed by nutrition or health websites and diet or health books. This suggests that despite the popularity of certain sources, when it comes to applying information to one’s diet, participants were more discerning, favoring sources that are more closely linked to science and expert advice.

Participants possessing fewer books were more likely to rely on lower quality sources for making dietary changes. This pattern might reflect disparities in access to quality information or differences in information literacy skills. The literature provides compelling evidence that lower literacy skills predict susceptibility to health misinformation [19,36], which in turn could mislead decision making and increase the risk of health harm [37]. Therefore, efforts to improve access to high-quality information sources and enhance critical appraisal skills could be beneficial in mitigating the risk of health harm associated with misinformed dietary decisions [1].

### 4.3. Source Trust, Not Use, Is a More Reliable Predictor of Subsequent Eating Behavior Changes

To the best of our understanding, no previous studies have addressed the question of whether seeking diet-nutrition information from sources of varying quality and the level of trust individuals place in these sources have an impact on individuals’ reliance on them for making dietary changes. Our results reveal that source trustworthiness plays a more prominent role than source-seeking behaviors in influencing dietary change decisions. Of the three different correlations investigated, the strongest was found between the level of trust in a source and reliance on it for dietary modifications. This suggests that a higher degree of trust may lead to greater reliance on the source for effecting dietary changes. Conversely, when a source is less trusted, there may be a reduced likelihood of relying on it for changing diet.

The correlation between source-seeking frequency and reliance for making dietary changes, though strong, was still less robust than the correlation observed between assigned source trustworthiness and reliance for making dietary changes. This highlights that the frequency of information-seeking behaviors may not necessarily translate into greater trust or reliance on a source for making dietary changes. This finding is encouraging because it presupposes that actively seeking information from inferior quality sources, which may potentially contain misinformation, may not always have a direct influence on dietary change decisions. Rather, it underscores the role of trustworthiness evaluations as a precursor to making informed choices.

Such a premise is reinforced by the moderate correlation between source-seeking frequency and level of trust, suggesting that frequent information seeking from a source does not seem to be a reliable predictor of source trustworthiness, and vice versa. This aligns with data from 54 empirical samples pointing to other factors besides trust as drivers of health-information-seeking behaviors, including ease of access, relevance, and utility [23]. As a case in point, in our sample, despite most respondents recognizing scientific journals and academic manuals as being trustworthy, information seeking from scholarly sources was very low. This disconnect could be attributed to multiple contextual barriers, such as the complex nature of the scientific literature, limited open access or high access costs, the need for specific search terms, and the skills and time required for analyzing, interpreting, and comparing content in scientific publications [1].

Further examination of the data revealed disparities in information seeking, trustworthiness, and reliance for making dietary changes, depending on the source type. For example, most respondents who frequently sought information from nutrition–health websites trusted and relied on these for making dietary changes, whereas those who infrequently or never sought information from this source type expressed more mistrust and a minimal level of reliance for their dietary change decisions. This may indicate a weaker discernment of nutrition–health websites as a poorer quality source by their more frequent seekers. A similar trust and change reliance pattern was observed among participants who frequently obtained diet-nutrition information from subpar sources such as social media, magazines, newspapers, diet/health books, and Google or Internet searches, possibly pointing to a diminished ability to discern source quality.

Conversely, higher quality sources such as scientific journals, academic manuals, and science news publications showed a different trend. Even though a substantial number of non-seekers and rare-seekers expressed trust in these sources, only a smaller proportion of them relied on these sources for making dietary changes. This incongruity reveals a disparity between greater levels of assigned trustworthiness and a reduced ability to obtain or distill information conductive of dietary change from scientifically dense sources. Accordingly, the focus on utility, accessibility, and comprehensibility/ease of understanding is indispensable alongside the cultivation of trust in the utilization of and reliance on scientific sources [14].

### 4.4. Strengths and Limitations

The present study benefits from a sizable sample size, which contributes to its strength, despite the moderate geographical, demographic (excluding age), and social diversity. Moreover, the web-based design of the study promoted accessibility and encouraged broad participation from diverse age groups and backgrounds.

One of the limitations of the study is the use of an opportunity sample. While convenient for recruitment purposes, it restricts the ability to generalize the findings to the broader population. Thus, our research should be regarded as exploratory in nature. Nevertheless, it is worth noting that the recruited sample size was substantial for this type of research, and the online survey design enabled us to capture qualitative data concurrently.

It is important to acknowledge that our study did not capture detailed information about specific changes in food and eating behaviors, or their nature, magnitude, or health effects. Instead, it explored the impact of each information source on the frequency of unspecified dietary changes reported by participants, providing a broader perspective on the overall influence of information sources on their eating behaviors. Notably, no other published study has simultaneously measured the complex interplay between information source-seeking frequencies, the level of trust in sources of varying quality, and the extent of self-reported dietary changes influenced by each source, making our research unique in its approach whilst providing a foundation for further investigations.

Another constraint of the study is that it did not refer to all of the evolving sources of diet-nutrition information and advice, such as AI-driven apps and devices [38], human-like AI chatbots like Nutripedia [39] and OpenAI’s Chat GPT [40,41,42], massive multiplayer online role-playing games (MMORPGs) [43,44], online courses [45,46], and other emerging digital platforms. While it was not feasible to explore every possible source, a strength of the study lies in its comprehensive coverage of 22 existing sources of diet-nutrition information and advice which more generically captured digital sub-types, allowing for a broad understanding of the landscape. These sources included various categories of online and digital information as well as interpersonal sources (such as nutrition and fitness professionals, medical doctors, nurses or health coaches, influencers, celebrity experts, famous personalities), recognizing the significance of both online and personal interactions in accessing diet-nutrition information.

Item non-response bias is a commonly observed limitation in online survey studies, as skipped questions may affect the completeness of the data available for analysis [47]. However, this survey exhibited a high completion rate of 79.2%, with an average of 711 out of 3419 skipped responses per question (based on the 16 non-compulsory questions presented after obtaining consent). The presence and complexity of matrix-style questions in the survey may have contributed to item non-response, as these are known to demand more effort and consideration than standard item-by-item questions [48]. Another recognized limitation in survey research is the occurrence of speeding [49], where respondents may engage in fast random clicking. Nonetheless, the average time taken by participants to complete the survey was 8 min and 19 s, suggesting that such behavior was relatively rare.

The respondents’ level of education and awareness about the characteristics of the recruited population (i.e., students and subscribers of The Health Sciences Academy) might have influenced their expectations of health and nutrition evidence-based knowledge and their skepticism towards lower quality information sources. However, we believe that this is unlikely to have significantly affected response bias since anonymous web-based questionnaires, like the one used in our study, typically mitigate social desirability bias (i.e., fear of disapproval) and encourage more honest reporting of sensitive information [50,51,52]. This is further supported by the findings demonstrating a discrepancy between the popularity and the perceived trustworthiness of different sources of diet-nutrition information, where nutrition–health websites, Google–Internet searches, and diet–health books were identified as the most commonly consulted sources, although participants did not place the highest level of trust in these.

In respect to the qualitative analysis, it should be acknowledged that several responses to the open-ended question encompassed multiple themes, reflecting the intricate and multifaceted nature of participants’ perceptions of trustworthiness in diet-nutrition information or advice. This overlap of themes could potentially lead to an overestimation or underestimation of the prevalence of specific perspectives and trustworthiness judgements. Additionally, the complex nature of participants’ perceptions may not be fully captured by written responses without a dialogue and lengthier explanations; thus, further qualitative research is necessary to gain a more comprehensive understanding of people’s views on the trustworthiness of diet-nutrition information or advice.

Lastly, it is worth noting that our study does not conclusively establish causation, such as a definite cause-and-effect relationship between seeking subpar sources and making misinformed or misguided dietary decisions that are riskier to health. Rather, our findings suggest that individuals are more likely to base their dietary changes on poorer quality information sources when they trust these sources more.

## 5. Conclusions

The key implications of our study are twofold. Firstly, the lack of previous research addressing the relationship between seeking diet-nutrition information, trust in sources of varying quality, and reliance on these sources for making dietary changes highlights a gap in the current knowledge base. Our study acts as a starting point in addressing this gap by providing insights into the dynamics between these variables.

Secondly, our findings reveal that the assigned trustworthiness of information sources exerts greater influence on the reliance on these sources for making dietary changes compared to active source seeking or utilization. This implies that individuals’ level of trust in a particular source may significantly impact their decision to rely on that source when making dietary modifications. The stronger the trust in a source, the higher the likelihood of relying on it for changing eating behaviors. Conversely, when trust in a source is lower, individuals may be less inclined to rely on it for altering their diet, as shown by our empirical data. Future research should delve deeper into the mechanisms that contribute to trust-building in higher quality diet-nutrition information sources, as well as explore strategies aimed at improving the quality of diet-nutrition information sources, ultimately facilitating safer and better-informed decision making in dietary changes.

The qualitative analysis further reinforced the importance of trustworthiness. Participants favored evidence-based information, which they associated with scientific backing and verifiability. They also highly valued practical yet truthful information from reputable sources and professionals in the field. While source trustworthiness seemed to play a role in source reliance for effecting dietary changes, so did practicality—a factor highlighted by approximately one in five qualitative responses. Also, the most trusted sources were not necessarily the most relied upon for changing eating behaviors, where scientific journals and academic manuals were ranked highly on trustworthiness scores but lower on reliance for change. Therefore, enhancing accessibility to credible, evidence-based nutrition information that is also engaging, easy-to-understand, and applicable to the individual’s context could foster tighter integration between practical information-seeking behaviors and trustworthiness evaluations.

Encouragingly, our findings demonstrate that higher quality sources had a greater influence on modifying eating behavior, while inferior quality sources resulted in fewer self-reported dietary changes. Despite the popularity of certain sources, participants were more discerning, favoring scientifically driven sources for guiding their dietary changes. That said, we also found that lower levels of general literacy may contribute to increased reliance on easily accessible but potentially misleading sources, such as social media, for obtaining nutrition information and modifying one’s diet. Promoting literacy and encouraging individuals to seek higher quality sources of nutrition information are crucial in mitigating the risks of misinformation, disinformation, and malinformation accompanying subpar sources. Furthermore, efforts to enhance critical thinking skills, including misinformation risk assessment skills, can empower individuals to evaluate the credibility and safety of nutrition information encountered on digital platforms and elsewhere.

These insights accentuate the importance of recognizing and encouraging credible, evidence-based nutrition information sources to support better informed and safer dietary change decisions, ultimately mitigating the potential for adverse health outcomes. Further studies are warranted to elucidate these complex relationships and inform effective misinformation discernment strategies and misinformation risk assessment frameworks.

## Figures and Tables

**Table 1:** Self-reported diet-nutrition information-seeking frequency per source.

		Information-Seeking Frequency per Source						
Ranking of Sources from Most to Least Sought-After		Never	Rarely	Sometimes	Often	Always	WAVG *	Total Responses *n*
		%						
1	Nutrition or health websites	4.2	8.2	34.6	39.4	13.6	2.50	2660
2	Google or Internet searches	6.1	11.0	34.0	34.7	14.2	2.40	2647
3	Diet or health books	8.5	13.4	37.7	29.9	10.5	2.20	2657
4	Scientific journals or academic manuals	19.3	17.8	30.3	22.4	10.2	1.86	2640
5	Science news publications	17.5	17.9	34.9	21.6	8.3	1.85	2639
6	Nutrition scientists and PhDs	25.9	14.7	26.9	22.0	10.5	1.76	2650
7	A nutrition professional	23.5	17.2	29.3	19.4	10.6	1.76	2652
8	Social media	30.5	23.7	29.4	12.7	3.7	1.35	2637
9	Film or TV documentaries	26.9	26.7	33.5	11.0	1.9	1.34	2635
10	Blogs or podcasts	31.4	22.6	29.7	13.0	3.3	1.34	2619
11	Friends, colleagues, or peers	27.6	26.6	33.3	10.4	2.1	1.33	2640
12	Online groups or forums	32.0	23.7	28.4	12.7	3.2	1.31	2631
13	Magazines or newspapers	29.9	27.7	31.4	8.7	2.2	1.26	2642
14	Influencers I follow on social media	39.5	19.1	25.5	12.1	3.8	1.22	2641
15	Family members	38.3	27.7	23.5	7.8	2.7	1.09	2635
16	A nurse or health coach	44.7	20.4	23.1	8.7	3.2	1.05	2637
17	My own medical doctor or GP	44.8	24.5	20.3	7.4	3.0	0.99	2645
18	Celebrity doctors or experts	47.1	20.3	21.9	8.0	2.7	0.99	2640
19	School, college, or university teachers or lecturers	51.4	17.8	18.9	8.6	3.4	0.95	2637
20	Gym instructors or personal trainers	49.3	21.2	19.8	7.2	2.4	0.92	2642
21	Famous personalities, actors, or presenters	69.0	17.3	9.7	2.7	1.3	0.50	2643
22	Fiction books or movies	76.7	13.0	7.1	2.0	1.3	0.38	2630

* The ranking of sources from highest to lowest information-seeking behavior frequencies is based on weighted averages per source calculated on a scale comprising ‘never’ (0), ‘rarely’ (1), ‘sometimes’ (2), ‘often’ (3), and ‘always’ (4).

**Table 2:** Diet-nutrition information trustworthiness level attributed to each source.

		Assigned Trustworthiness Level to Each Source						
Ranking of Sources from Most to Least Trusted		Least Trustworthy	Not Very Trustworthy	Trustworthy	Very Trustworthy	Most Trustworthy	WAVG *	Total Responses *n*
		%						
1	Nutrition scientists and PhDs	1.7	4.4	32.6	31.1	30.1	2.84	2632
2	A nutrition professional	1.5	5.5	37.2	33.1	22.6	2.70	2630
3	Scientific journals or academic manuals	3.1	7.0	39.9	29.0	21.0	2.58	2608
4	Science news publications	3.9	10.5	46.5	26.8	12.4	2.33	2608
5	Nutrition or health websites	2.5	16.5	54.8	19.9	6.3	2.11	2603
6	Diet or health books	3.1	22.3	52.4	16.4	5.8	2.00	2605
7	A nurse or health coach	6.3	21.7	52.6	15.1	4.3	1.89	2615
8	My own medical doctor or GP	12.0	27.3	42.1	13.2	5.5	1.73	2616
9	School, college, or university teachers or lecturers	11.7	28.7	44.1	11.9	3.6	1.67	2606
10	Google or Internet searches	8.7	43.4	39.1	6.4	2.4	1.50	2599
11	Gym instructors or personal trainers	12.7	39.1	39.0	7.4	2.0	1.47	2621
12	Film or TV documentaries	20.4	36.3	37.5	5.0	0.9	1.30	2590
13	Blogs or podcasts	19.0	43.4	32.4	4.5	0.7	1.25	2579
14	Celebrity doctors or experts	24.8	36.7	30.6	6.2	1.8	1.24	2610
15	Family members	23.4	42.2	28.4	4.3	1.7	1.19	2608
16	Friends, colleagues, or peers	22.3	46.4	27.7	3.0	0.7	1.13	2606
17	Online groups or forums	21.3	49.1	25.5	3.4	0.7	1.13	2584
18	Magazines or newspapers	24.9	46.8	24.5	2.9	0.9	1.08	2600
19	Influencers I follow on social media	37.4	39.1	19.3	3.0	1.2	0.92	2604
20	Social media	39.1	45.7	12.7	1.9	0.6	0.79	2595
21	Famous personalities, actors, or presenters	54.8	36.1	7.3	1.3	0.5	0.56	2615
22	Fiction books or movies	67.7	23.7	7.0	1.0	0.5	0.43	2593

* The ranking of sources from highest to lowest levels of assigned trustworthiness is based on weighted averages per source calculated on a scale comprising ‘least trustworthy’ (0), ‘not very trustworthy’ (1), ‘trustworthy’ (2), ‘very trustworthy’ (3), and ‘most trustworthy’ (4).

**Table 3:** Self-reported eating behavior change frequency per information source.

		Frequency of Eating Behavior Changes per Source						
Ranking of Sources from Most to Least Relied-Upon for Making Dietary Changes		Never	Rarely	Sometimes	Often	All the Time	WAVG *	Total Responses *n*
		%						
1	A nutrition professional	12.4	10.5	39.2	27.0	10.9	2.13	2436
2	Nutrition scientists and PhDs	13.2	11.7	39.4	25.7	10.1	2.08	2428
3	Nutrition or health websites	7.7	17.4	49.5	20.3	5.2	1.98	2424
4	Diet or health books	10.8	17.5	46.4	20.7	4.7	1.91	2416
5	Scientific journals or academic manuals	17.0	17.7	39.0	19.7	6.5	1.81	2408
6	Science news publications	18.6	20.0	40.9	15.8	4.8	1.68	2409
7	Google or Internet searches	19.0	27.1	39.3	11.4	3.1	1.53	2417
8	A nurse or health coach	33.0	21.2	32.1	11.0	2.7	1.29	2426
9	My own medical doctor or GP	34.4	22.4	27.8	11.2	4.2	1.29	2431
10	Family members	37.8	28.7	26.5	5.3	1.7	1.05	2433
11	Gym instructors or personal trainers	43.0	23.4	24.1	7.2	2.3	1.02	2427
12	Friends, colleagues, or peers	38.0	31.6	25.6	3.5	1.2	0.98	2429
13	Film or TV documentaries	41.1	27.4	24.7	5.8	1.0	0.98	2416
14	School, college, or university teachers or lecturers	46.2	23.7	21.8	6.7	1.6	0.94	2427
15	Blogs or podcasts	44.6	25.5	23.0	5.8	1.1	0.93	2404
16	Magazines or newspapers	43.3	30.1	21.9	3.7	1.0	0.89	2412
17	Online groups or forums	46.8	27.1	21.3	3.8	1.0	0.85	2413
18	Celebrity doctors or experts	52.6	22.4	18.8	4.7	1.6	0.80	2425
19	Influencers I follow on social media	53.1	21.9	19.0	4.7	1.3	0.79	2431
20	Social media	51.5	25.7	17.8	3.8	1.2	0.77	2414
21	Famous personalities, actors, or presenters	70.4	18.5	8.6	1.7	0.8	0.44	2429
22	Fiction books or movies	81.0	11.6	5.4	1.3	0.7	0.29	2412

* The ranking of sources used from highest to lowest eating behavior change frequencies is based on weighted averages per source calculated on a scale comprising ‘never’ (0), ‘rarely’ (1), ‘sometimes’ (2), ‘often’ (3), and ‘all the time’ (4).

## Data Availability

Data are available from the corresponding author upon reasonable request.

## Supplementary Materials

The following supporting information can be downloaded at https://www.mdpi.com/article/10.3390/nu15214515/s1: Table S1: General characteristics of the study participants; Table S2: Sources of nutrition information and dietary advice listed in the survey questionnaire; Table S3: Participants’ own interpretation of/viewpoints about trustworthy diet-nutrition information or advice. Table S4: Cross-tabulations of self-reported diet-nutrition information source-seeking frequencies with levels of trust and reliance for making dietary changes. Table S5: Comparison of dietary change frequencies per each of six subpar information sources relative to the number of books owned.

## Abbreviations

WAVG—weighted average. *r_s_*—Spearman correlations. AI—artificial intelligence.
