# Supplementary material for: Diet-Nutrition Information Seeking, Source Trustworthiness, and Eating Behavior Changes: An International Web-Based Survey
Source: Nutrients. 2023 Oct 25;15(21):4515. doi: 10.3390/nu15214515 (PMC10649819; doi:10.3390/nu15214515)
Supplement: Supplementary file 1 [file nutrients-15-04515-s001.zip › nutrients-2647282-supplementary/Supplementary Table S1_Ruani Reiss Kalea.pdf]

nutrients

MDPI

Table S1. General characteristics of the study participants.

	Variables	*n*	%
**Age**	18-20	80	2.5
	21-25	291	9.2
	26-30	331	10.4
	31-35	403	12.7
	36-40	376	11.8
	41-45	399	12.5
	46-50	339	10.7
	51-55	359	11.3
	56-60	258	8.1
	61-65	195	6.1
	66-70	93	2.9
	70 or older	58	1.8
**Gender**	Male	665	21.0
	Female	2477	78.2
	Other	5	0.2
	Prefer not to say	19	0.6
**Employment status**	Employed, working 40 or more hours per week	950	31.0
	Employed, working 1-39 hours per week	1171	38.2
	Not employed, looking for work	373	12.2
	Not employed, NOT looking for work	259	8.4
	Retired	236	7.7
	Disabled, not able to work	80	2.6
**Country of residence**	United Kingdom	1130	35.9
	United States	484	15.4
	Canada	206	6.5
	Australia	177	5.6
	India	177	5.6
	Ireland	69	2.2
	Nigeria	54	1.7
	South Africa	54	1.7
	Egypt	50	1.6

	Variables	*n*	%
	Pakistan	43	1.4
	New Zealand	32	1.0
	Spain	29	0.9
	Philippines	26	0.8
	Kenya	24	0.8
	Malaysia	23	0.7
	France	20	0.6
	Bangladesh	19	0.6
	United Arab Emirates	19	0.6
	Ethiopia	18	0.6
	Italy	17	0.5
	Mexico	16	0.5
	Netherlands	14	0.4
	Russian Federation	14	0.4
	Germany	13	0.4
	Romania	13	0.4
	Singapore	13	0.4
	Ghana	11	0.4
	Ukraine	11	0.4
	Hungary	10	0.3
	Lebanon	10	0.3
	Uganda	10	0.3
	Cyprus	9	0.3
	Greece	9	0.3
	Indonesia	9	0.3
	Iran	9	0.3
	Portugal	9	0.3
	Saudi Arabia	9	0.3
	Other 100 countries	290	9.2
**Physical and digital books owned**	0-9	346	10.9
	10-99	1204	37.9
	100-499	1102	34.7
	500-999	341	10.7
	1000 or more	183	5.8

	Variables	*n*	%
**Popular diets tried in the past**	Low-carb	1357	44.2
	Low-calorie	1282	41.7
	Low-fat	1169	38.0
	Intermittent fasting	1144	37.2
	Vegetarian	1132	36.8
	High-protein	1084	35.3
	Plant-based	880	28.6
	Dairy-free	869	28.3
	Gluten-free	811	26.4
	Mediterranean	708	23.0
	Juicing	651	21.2
	Vegan	630	20.5
	Ketogenic	616	20.1
	Time-restricted eating	611	19.9
	Low-glycaemic-index	503	16.4
	Meat-based	417	13.6
	Pescatarian	303	9.9
	Low-FODMAP	196	6.4
	DASH	110	3.6
	Other	401	13.1
**Current dietary pattern**	I only eat animal foods	9	0.3
	I only eat plant foods	306	9.8
	I predominantly eat plant foods, plus some animal foods	984	31.5
	I predominantly eat animal foods, plus some plant foods	261	8.4
	I eat a balance of plant foods and animal foods	1562	50.0
**Self-reported health**	Very poor	27	1.1
	Poor	107	4.2
	Somewhat good	520	21.5
	Good	1180	48.8
	Great	585	24.2
**Self-reported body weight categorization**	Obese	154	6.3
	Overweight	319	13.0
	Slightly overweight	556	22.6

	Variables	*n*	%
	Normal weight	1271	51.8
	Slightly underweight	136	5.5
	Underweight	20	0.8
**Self-reported physical activity levels**	Sedentary	239	9.7
	Moderately active	955	38.8
	Active	866	35.2
	Very active	400	16.3
**Interest in nutrition and health**	Extremely interested	1535	62.7
	Very interested	697	28.5
	Interested	206	8.4
	Not very interested	10	0.4
	Not interested	2	0.1
**Being a nutrition professional**	Yes	436	17.9
	No	1438	58.9
	Studying for it	566	23.2
