# Supplementary material for: Diet-Nutrition Information Seeking, Source Trustworthiness, and Eating Behavior Changes: An International Web-Based Survey
Source: Nutrients. 2023 Oct 25;15(21):4515. doi: 10.3390/nu15214515 (PMC10649819; doi:10.3390/nu15214515)
Supplement: Supplementary file 1 [file nutrients-15-04515-s001.zip › nutrients-2647282-supplementary/Supplementary Table S2_Ruani Reiss Kalea.pdf]

nutrients

MDPI

Table S2. Sources of nutrition information and dietary advice listed in the survey questionnaire.

Interpersonal Sources of Information	General Sources of Information
A nurse or health coach	Blogs or podcasts
A nutrition professional	Diet or health books
Celebrity doctors or experts	Fiction books or movies
Family members	Film or TV documentaries
Friends, colleagues or peers	Google or Internet searches
Gym instructors or personal trainers	Magazines or newspapers
Influencers I follow on social media	Social media
My own medical doctor or GP	Nutrition or health websites
School, college or university teachers or lecturers	Online groups or forums
Famous personalities, actors or presenters	Scientific journals or academic manuals
Nutrition scientists and PhDs	Science news publications
