# Supplementary material for: Diet-Nutrition Information Seeking, Source Trustworthiness, and Eating Behavior Changes: An International Web-Based Survey
Source: Nutrients. 2023 Oct 25;15(21):4515. doi: 10.3390/nu15214515 (PMC10649819; doi:10.3390/nu15214515)
Supplement: Supplementary file 1 [file nutrients-15-04515-s001.zip › nutrients-2647282-supplementary/Supplementary Table S3_Ruani Reiss Kalea.pdf]

nutrients

Table S3. Participants' own interpretation of/viewpoints about trustworthy diet-nutrition information or advice.

	Descriptive Themes of Trustworthy Diet-Nutrition Information / Advice	Coding Keywords (Including Typos and Variations)	Examples of Responses	*n* Coded Observations	*n* Responses	% of Responses *
**1**	Evidence-based	evidence, research, science, scientific	*"Science-based, predominantly non-biased (the person giving the advice does not WANT something to be true, rather follows scientific evidence), non-radical, always cautious (the person giving advice does not talk as if he/she owns the ABSOLUTE truth), non-trendy."*<br><br>*"TRUSTWORTHY nutrition information/ dietary advice means that the information or advice is strongly scientifically based or come from the combination of several peer review studies, which took place for a long period and involving many people."*	1084	779	44.7
**2**	With references to research or verifiable sources	reference, studies, citation, sourced, verifiable	*"When the information is provided on a website, there should be references to the studies where this has been proven."*<br><br>*"I like references. If I can read a book and there is a direct link to the researched topic that is more real for me."*	514	396	22.7
**3**	From a familiar, credible, or reputable source	credible, professional, reputable, reputed, familiar, reliable source, authority, credibility, qualified, certified, expert, opinion, expertise, qualification, PhD	*"If looking at research, peer reviewed studies are always preferred. Trustworthy information otherwise would be obtained through sources that I am familiar with and have some background knowledge of the education or sources that are being used."*<br><br>*"It means it comes from someone with authority as opposed to someone who for example was overweight, did the diet and now they're an expert."*	389	324	18.6
**4**	Practical and applicable	practical, applicable, realistic, feasible, accessible, results, long-term, sustainable, success,	*"Advice that does not require you to buy products. Advice that is practical and sensible – not extreme."*<br><br>*"Something that is realistic. I know there is no quick fix. It has to be*	345	299	17.1

		proven methods, tried and tested, personal experience, proven to work	*something I can stick with and normal food that I like."*			
**5**	Truthful, accurate, and reliable	objective, accurate, true, truthful, honest, transparent, reliable information, factual	*"We all have an opinion & rationale as to what we prefer to eat & why. Some of us may want others to abide by our dietary beliefs as well, however I believe that providing honest, unbiased, fact telling [information] is of utmost importance whenever educating anyone about health/nutrition so that one is aware of their options & can then make an informed choice that speaks to them."* *"A person or institution with a reputation for honesty and great research."*	207	186	10.7
**6**	Unbiased	bias, industry, pharma, impartial, declaration, funded, pharma, commercial	*"Information that is based on the balance of the best available scientific evidence, by a person or organization that is free from any bias, or has at least declared any conflicts of interests/sources of funding."* *"Trustworthy means doing my own research thoroughly due to hidden agendas ie: food/pharmaceutical companies/television/ media not giving truthful or unbiased information."*	175	131	7.5
**7**	Established	time-tested, period of time, over time, current, recent	*"Something that's been proven to work over a longer period of time.... years not months or days."* *"I only trust it if the information is backed by longevity. I need to know that the way of eating has been around for a long time with proven results."*	156	123	7.1
**8**	Peer-reviewed or verified	peer-reviewed, verified information, moderation, journal, high-impact	*"Based on peer-reviewed studies in professional journals, or books/papers written by academics."* *"It must be peer reviewed and originally appear in a scientific journal, and have it's data be repeatable. It can also be summarised by a practicing professional."*	193	113	6.5

**9**	Personalized	personalized, individualized, customized, tailored, relevant advice	*"Personalised, to your circumstances, by an expert who knows you and your needs/issues, etc., as standardised advice may not be suitable."*<br><br>*"Nutrition information that isn't going to harm me if I follow it. Preferably something tailored to me in some way and not just arbitrary 'one size fits all' advice."*	81	72	4.1
**10**	Complete with strengths and limitations	comprehensive, complete, pros, cons, benefits, risks	*"Science based information that looks at both the pros and cons of the topic at hand which allows the individual to make an informed decision."*<br><br>*"Advice from a source which seems to have done extensive research, and presents pros and cons to their advice, as well as pros and cons to the alternatives."*	71	62	3.6
**11**	Rational and explanatory	rational, logical explanation, reason, education	*"It is based on LOGICAL Explanation and proved data."*<br><br>*"1. Not sponsored by big food companies; 2. Some scientific explanation accompanying the nutritional advice; 3. Backed by research"*	59	53	3.0
**12**	Void of fads and miracle solutions	fad, trend, miracle, magic, pseudo	*"From a reliable source (no 'influencers' or celebrities touting the latest miracle bit of tat), with data and results to back it up. Trustworthy information doesn't offer a miracle. Also trustworthy means that I have done my own research and read around the subject to ensure I'm taking in a wide range of views (all from reliable sources). Lots of what we read or see on the subject has a bias of some kind in order to make it a story that people want to spend time with."*<br><br>*"Trustworthy nutrition information/advice means it is; 1) NOT government dictated as those recommendations are generally driven by the dairy and meat industries 2) efficacious, proven using studies over a period of time, 3) NOT a fad or quick-fix style of eating i.e. cabbage soup diets or others which require*	55	50	2.9

		removal of food groups for extended periods of time in order to drop weight fast/quickly."			
**13** Based on significant findings or large samples	large, big sample, power, meta, effect size, significant	*"Science-based empirical metadata that has been exhaustively peer-reviewed over a significant period of time, robust control baseline data & premises that support discovery rather than a specific outcome. Broadness and volume of test subjects, sample range and size." #31*<br><br>*"Research that has been done on larger sample sizes and been repeated (not just one small research study on a small sample size)."*	49	46	2.6
**14** Simple and specific	concise, simple, simplicity, specific, clear, understandable, jargon-free	*"Authentic, simple and shortly described that I feel that is for my best benefit taking into account my health status and personality. Information that has been researched uniquely and has value of the professional team that follow their life passion about being healthy."*<br><br>*"Advice that is based on solid evidence which is jargon free and is available to be utilised by all, regardless of income or level of education."*	43	38	2.2

* Percentages calculated using 1744 responses in total. Note that a single response may fall under one or more descriptive themes.
