# Supplementary material for: Diet-Nutrition Information Seeking, Source Trustworthiness, and Eating Behavior Changes: An International Web-Based Survey
Source: Nutrients. 2023 Oct 25;15(21):4515. doi: 10.3390/nu15214515 (PMC10649819; doi:10.3390/nu15214515)
Supplement: Supplementary file 1 [file nutrients-15-04515-s001.zip › nutrients-2647282-supplementary/Supplementary Table S4_Ruani Reiss Kalea.pdf]

nutrients

MDPI

Table S4. Cross-tabulations of self-reported diet-nutrition information source-seeking frequencies with levels of trust and with reliance for making dietary changes.

Frequency of Nutrition-Diet Information Source-Seeking Behaviors		Assigned Level of Trust Relative to Source-Seeking Frequency						Reliance for Making Dietary Changes Relative to Source-Seeking Frequency					
		Least Trustworthy	Not Very Trustworthy	Trustworthy	Very Trustworthy	Most Trustworthy	Total Responses	Never	Rarely	Sometimes	Often	All the Time	Total Responses
		%*					*n*	%*					*n*
**Nutrition or health websites**	Never	**24.8**	**33.7**	**35.6**	**3.0**	**3.0**	101	**54.8**	**20.4**	**22.6**	**1.1**	**1.1**	93
	Rarely	**6.7**	**36.4**	**42.1**	**12.4**	**2.4**	209	**19.5**	**39.5**	**36.8**	**3.2**	**1.1**	190
	Sometimes	**1.6**	**24.0**	**60.8**	**11.2**	**2.5**	893	**6.7**	**25.5**	**58.2**	**8.0**	**1.7**	827
	Often	**0.7**	**8.4**	**59.2**	**26.5**	**5.2**	1035	**3.4**	**10.5**	**52.5**	**30.4**	**3.3**	978
	Always	**1.2**	**4.7**	**38.7**	**32.6**	**23.0**	344	**2.5**	**3.8**	**32.4**	**37.1**	**24.1**	315
**Google or Internet searches**	Never	**50.6**	**43.0**	**5.1**	**0.0**	**1.3**	156	**84.8**	**11.3**	**3.3**	**0.0**	**0.7**	151
	Rarely	**20.2**	**63.1**	**14.9**	**1.1**	**0.7**	282	**39.1**	**43.3**	**16.1**	**1.5**	**0.0**	261
	Sometimes	**6.3**	**56.2**	**35.5**	**1.6**	**0.5**	876	**7.0**	**18.3**	**56.4**	**16.6**	**1.8**	833
	Often	**2.3**	**34.4**	**53.2**	**8.4**	**1.6**	900	**5.2**	**10.0**	**37.4**	**31.6**	**15.8**	329
	Always	**2.0**	**18.8**	**48.2**	**20.2**	**10.9**	357	**17.6**	**40.9**	**37.2**	**3.4**	**0.9**	814
**Diet or health books**	Never	**19.7**	**44.6**	**31.0**	**2.8**	**1.9**	213	**58.9**	**21.4**	**16.2**	**2.1**	**1.6**	192
	Rarely	**3.7**	**40.2**	**47.6**	**7.1**	**1.4**	351	**22.2**	**40.6**	**32.8**	**3.4**	**1.0**	320
	Sometimes	**1.8**	**24.4**	**59.1**	**12.7**	**2.0**	974	**6.5**	**20.1**	**60.8**	**11.4**	**1.2**	898
	Often	**0.3**	**11.7**	**58.1**	**24.2**	**5.7**	776	**1.2**	**7.7**	**48.0**	**38.6**	**4.5**	741
	Always	**2.3**	**3.8**	**34.5**	**30.7**	**28.8**	267	**2.5**	**2.9**	**30.7**	**37.3**	**26.6**	241
**Scientific journals or academic manuals**	Never	**9.9**	**13.7**	**45.6**	**18.6**	**12.3**	496	**55.5**	**22.9**	**17.7**	**3.0**	**0.9**	436
	Rarely	**3.5**	**10.5**	**45.4**	**27.9**	**12.7**	456	**19.1**	**33.8**	**35.7**	**10.2**	**1.2**	420
	Sometimes	**0.8**	**5.4**	**46.6**	**32.4**	**15.0**	782	**8.5**	**18.2**	**52.5**	**17.8**	**3.0**	730
	Often	**0.9**	**2.3**	**31.1**	**37.4**	**28.4**	578	**2.6**	**6.6**	**44.8**	**37.8**	**8.2**	547
	Always	**1.2**	**2.3**	**18.8**	**24.9**	**52.9**	261	**2.5**	**3.3**	**31.2**	**30.7**	**32.4**	244
**Science news publications**	Never	**11.7**	**21.5**	**45.7**	**15.0**	**6.1**	446	**56.0**	**22.3**	**18.3**	**2.3**	**1.3**	400
	Rarely	**5.7**	**14.4**	**55.2**	**20.1**	**4.6**	458	**22.8**	**36.0**	**34.8**	**6.1**	**0.2**	425
	Sometimes	**1.6**	**8.8**	**53.6**	**27.3**	**8.8**	901	**12.1**	**20.2**	**51.0**	**14.4**	**2.2**	825
	Often	**1.1**	**2.7**	**38.2**	**40.5**	**17.6**	558	**2.5**	**9.3**	**51.9**	**30.1**	**6.3**	528
	Always	**1.4**	**5.2**	**21.8**	**28.4**	**43.1**	211	**4.5**	**7.5**	**29.2**	**30.7**	**28.1**	199
	Never	**4.8**	**7.7**	**39.1**	**25.4**	**23.0**	673	**35.8**	**18.3**	**32.5**	**8.9**	**4.5**	606

**Nutrition scientists and PhDs**	Rarely	**1.3**	**7.3**	**39.8**	**30.1**	**21.5**	382	**11.9**	**20.1**	**45.6**	**17.9**	**4.5**	353
	Sometimes	**0.6**	**4.0**	**38.9**	**34.0**	**22.6**	705	**6.3**	**10.1**	**54.4**	**23.7**	**5.5**	651
	Often	**0.4**	**0.5**	**22.5**	**39.6**	**37.0**	573	**1.7**	**4.8**	**36.3**	**43.8**	**13.6**	546
	Always	**0.7**	**1.1**	**12.2**	**20.7**	**65.2**	270	**2.1**	**1.2**	**16.5**	**44.0**	**36.2**	243
**A nutrition professional**	Never	**3.8**	**10.5**	**43.2**	**26.0**	**16.5**	101	**36.2**	**17.8**	**32.7**	**9.5**	**3.8**	550
	Rarely	**1.6**	**7.6**	**42.9**	**31.6**	**16.4**	74	**11.8**	**18.0**	**49.4**	**16.3**	**4.6**	417
	Sometimes	**0.7**	**3.9**	**41.7**	**33.9**	**19.8**	151	**5.8**	**8.6**	**50.5**	**28.9**	**6.2**	707
	Often	**0.4**	**2.0**	**28.8**	**44.0**	**24.9**	126	**1.5**	**2.9**	**33.9**	**47.1**	**14.7**	484
	Always	**1.1**	**2.6**	**17.1**	**30.1**	**49.1**	132	**1.6**	**0.8**	**14.2**	**41.3**	**42.1**	247
**Social media**	Never	**75.3**	**23.6**	**0.9**	**0.1**	**0.1**	780	**90.3**	**7.7**	**1.6**	**0.1**	**0.3**	730
	Rarely	**39.0**	**55.1**	**5.7**	**0.2**	**0.0**	613	**57.9**	**33.9**	**7.2**	**0.9**	**0.2**	570
	Sometimes	**17.3**	**61.5**	**19.8**	**1.2**	**0.3**	753	**26.9**	**39.2**	**30.0**	**3.1**	**0.9**	689
	Often	**11.0**	**49.7**	**33.1**	**5.5**	**0.6**	326	**13.0**	**27.7**	**44.0**	**14.0**	**1.3**	307
	Always	**5.8**	**25.6**	**34.9**	**22.1**	**11.6**	86	**10.1**	**8.9**	**36.7**	**25.3**	**19.0**	79
**Film or TV documentaries**	Never	**49.5**	**35.1**	**14.5**	**0.9**	**0.0**	683	**79.2**	**13.7**	**6.5**	**0.3**	**0.3**	629
	Rarely	**16.4**	**51.2**	**30.5**	**1.6**	**0.3**	691	**44.9**	**40.5**	**13.2**	**1.1**	**0.3**	637
	Sometimes	**7.1**	**32.9**	**54.1**	**5.5**	**0.4**	856	**20.8**	**32.3**	**40.4**	**6.1**	**0.5**	803
	Often	**3.2**	**14.2**	**59.9**	**19.2**	**3.6**	282	**8.9**	**16.7**	**47.0**	**25.2**	**2.2**	270
	Always	**2.1**	**21.3**	**38.3**	**21.3**	**17.0**	47	**9.1**	**6.8**	**31.8**	**27.3**	**25.0**	44
**Blogs or podcasts**	Never	**46.6**	**43.4**	**9.3**	**0.5**	**0.3**	788	**88.4**	**9.2**	**1.8**	**0.3**	**0.3**	717
	Rarely	**12.8**	**58.3**	**27.0**	**1.6**	**0.4**	571	**48.2**	**37.5**	**12.0**	**2.0**	**0.4**	544
	Sometimes	**4.1**	**43.1**	**48.3**	**4.1**	**0.4**	758	**18.9**	**36.0**	**40.4**	**3.7**	**1.0**	705
	Often	**1.5**	**22.9**	**59.0**	**15.4**	**1.2**	332	**5.1**	**19.0**	**51.0**	**23.4**	**1.6**	316
	Always	**4.9**	**17.3**	**43.2**	**24.7**	**9.9**	81	**8.0**	**18.7**	**26.7**	**34.7**	**12.0**	75
**Friends, colleagues or peers**	Never	**44.4**	**44.7**	**10.4**	**0.3**	**0.1**	709	**73.6**	**19.2**	**6.4**	**0.5**	**0.3**	660
	Rarely	**21.7**	**57.3**	**19.5**	**1.6**	**0.0**	683	**37.0**	**44.0**	**18.2**	**0.6**	**0.2**	638
	Sometimes	**9.9**	**46.5**	**40.4**	**2.6**	**0.6**	858	**19.4**	**35.8**	**40.7**	**3.0**	**1.0**	798
	Often	**6.7**	**27.9**	**49.1**	**13.4**	**3.0**	269	**10.3**	**23.3**	**44.7**	**18.2**	**3.6**	253
	Always	**9.3**	**20.4**	**50.0**	**14.8**	**5.6**	54	**12.2**	**18.4**	**34.7**	**16.3**	**18.4**	49
**Online groups or forums**	Never	**46.1**	**46.3**	**6.9**	**0.5**	**0.1**	807	**83.6**	**12.7**	**2.8**	**0.9**	**0.0**	743
	Rarely	**16.4**	**61.2**	**19.5**	**2.6**	**0.3**	610	**49.8**	**37.9**	**10.9**	**1.4**	**0.0**	570
	Sometimes	**7.2**	**52.1**	**37.4**	**2.9**	**0.4**	725	**23.1**	**37.2**	**36.7**	**2.4**	**0.6**	675
	Often	**3.7**	**33.5**	**52.4**	**8.8**	**1.5**	328	**10.8**	**23.1**	**51.0**	**13.6**	**1.6**	316
	Always	**5.4**	**17.6**	**44.6**	**21.6**	**10.8**	74	**16.2**	**11.8**	**26.5**	**25.0**	**20.6**	68
	Never	**55.1**	**36.1**	**7.8**	**0.8**	**0.3**	771	**81.6**	**14.5**	**3.5**	**0.3**	**0.1**	718

Magazines or newspapers	Rarely	19.1	63.3	15.9	1.7	0.1	713	42.6	42.9	13.2	1.1	0.2	650
	Sometimes	8.3	49.0	39.1	2.9	0.7	806	18.8	37.7	38.5	4.5	0.5	751
	Often	3.6	28.5	57.5	9.5	0.9	221	8.1	21.0	50.5	18.6	1.9	210
	Always	10.7	10.7	33.9	23.1	21.4	56	16.3	8.1	30.6	16.3	28.6	49
Influencers I follow on social media	Never	69.0	25.9	4.2	0.9	0.0	1016	85.6	10.3	3.5	0.4	0.1	939
	Rarely	27.2	55.0	16.8	0.8	0.2	493	50.8	31.3	14.8	2.6	0.6	467
	Sometimes	15.6	50.6	30.1	2.6	1.1	654	30.0	32.6	32.1	3.8	1.5	610
	Often	6.3	38.3	42.1	10.1	3.2	316	13.4	22.3	42.8	18.8	2.7	292
	Always	9.6	18.1	41.5	17.0	13.8	94	13.8	13.8	37.9	21.8	12.6	87
Family members	Never	48.0	41.1	9.7	1.0	0.3	984	72.0	20.2	6.6	0.7	0.5	925
	Rarely	13.6	58.1	26.0	2.0	0.4	716	27.9	47.3	22.1	2.2	0.5	673
	Sometimes	3.5	36.9	52.2	5.3	2.2	604	7.4	26.9	58.7	5.9	1.1	557
	Often	4.5	16.6	52.3	22.1	4.5	199	3.4	15.6	43.6	31.8	5.6	179
	Always	4.4	13.0	44.9	18.8	18.8	69	6.8	3.4	33.9	28.8	27.1	59
A nurse or health coach	Never	11.7	30.7	47.3	7.6	2.6	1143	55.7	21.6	19.3	2.5	0.9	1061
	Rarely	3.4	22.3	58.1	14.3	1.9	530	25.1	30.7	34.8	8.5	1.0	495
	Sometimes	1.2	12.6	62.1	20.3	3.9	597	10.0	17.9	53.1	17.0	2.0	548
	Often	1.3	5.3	47.8	38.5	7.1	226	10.1	9.7	36.2	35.3	8.7	207
	Always	2.5	6.2	32.1	23.5	35.8	81	4.0	5.3	26.3	35.5	29.0	76
My own medical doctor or GP	Never	22.8	35.2	32.5	7.4	2.1	1156	62.7	20.4	12.9	3.2	0.9	1071
	Rarely	5.6	32.7	49.5	9.4	2.8	640	18.9	38.6	35.1	6.2	1.2	593
	Sometimes	1.9	15.9	56.1	19.9	6.2	529	7.1	15.8	51.9	18.7	6.5	493
	Often	2.1	2.6	42.4	36.1	16.8	191	4.5	5.6	30.2	46.4	13.4	179
	Always	1.3	4.0	24.0	26.7	44.0	75	4.5	3.0	19.4	31.3	41.8	67
Celebrity doctors or experts	Never	40.0	36.8	19.8	2.7	0.7	1211	77.0	14.2	7.2	1.0	0.7	1124
	Rarely	16.7	52.7	26.1	3.8	0.8	528	44.7	35.8	15.6	3.0	0.8	494
	Sometimes	7.5	31.0	51.0	8.9	1.6	561	24.7	28.9	38.7	6.7	1.0	522
	Often	6.3	20.2	48.6	22.1	2.9	208	18.1	19.7	37.8	19.7	4.7	193
	Always	10.3	13.2	33.8	19.1	23.5	68	8.5	22.0	27.1	22.0	20.3	59
School, college or university teachers or lecturers	Never	19.2	38.4	34.8	6.1	1.6	1321	68.7	19.6	9.7	1.2	0.7	1223
	Rarely	5.3	29.0	55.0	8.7	2.0	458	33.6	37.1	24.7	4.4	0.2	434
	Sometimes	3.3	17.6	60.6	15.3	3.3	490	19.6	26.0	41.8	11.6	1.1	450
	Often	2.7	4.1	44.8	39.3	9.1	219	10.7	17.6	41.5	24.4	5.9	205
	Always	1.2	8.1	34.9	25.6	30.2	86	13.8	15.0	27.5	30.0	13.8	80
	Never	22.5	47.8	26.4	2.8	0.6	1271	71.8	18.4	8.5	1.0	0.3	1171

**Gym instructors or personal trainers**	Rarely	**4.4**	**45.7**	**44.2**	**4.8**	**0.9**	547	**28.4**	**42.6**	**25.3**	**2.9**	**0.8**	514
	Sometimes	**1.9**	**23.5**	**61.7**	**9.6**	**3.3**	519	**8.2**	**22.5**	**53.9**	**12.9**	**2.5**	488
	Often	**2.1**	**13.7**	**49.0**	**31.1**	**4.2**	190	**3.4**	**9.7**	**43.4**	**33.1**	**10.3**	175
	Always	**1.6**	**12.5**	**32.8**	**32.8**	**20.3**	64	**0.0**	**3.8**	**22.6**	**41.5**	**32.1**	53
**Famous personalities, actors or presenters**	Never	**67.6**	**29.1**	**3.0**	**0.3**	**0.1**	1789	**84.5**	**11.1**	**3.5**	**0.7**	**0.2**	1665
	Rarely	**33.9**	**54.9**	**9.4**	**1.1**	**0.7**	446	**47.0**	**38.2**	**12.9**	**1.2**	**0.7**	419
	Sometimes	**17.0**	**53.0**	**26.7**	**3.2**	**0.0**	247	**27.2**	**37.1**	**29.9**	**4.5**	**1.3**	224
	Often	**12.7**	**42.3**	**26.8**	**15.5**	**2.8**	71	**29.0**	**17.7**	**35.5**	**12.9**	**4.8**	62
	Always	**23.5**	**17.7**	**32.4**	**14.7**	**11.8**	34	25.9	14.8	14.8	22.2	22.2	27
**Fiction books or movies**	Never	**80.2**	**17.2**	**2.3**	**0.2**	**0.1**	1972	**92.3**	**5.5**	**1.9**	**0.2**	**0.1**	1843
	Rarely	**34.6**	**50.0**	**13.9**	**0.9**	**0.6**	330	**51.1**	**36.8**	**9.8**	**1.6**	**0.7**	307
	Sometimes	**16.5**	**41.5**	**35.2**	**5.7**	**1.1**	176	**32.3**	**30.3**	**25.8**	**8.4**	**3.2**	155
	Often	**10.2**	**46.9**	**34.7**	**4.1**	**4.1**	49	**31.8**	**20.5**	**31.8**	**15.9**	0.0	44
	Always	7.1	14.3	32.1	28.6	17.9	28	22.2	11.1	33.3	3.7	29.6	27

* Bolded values represent $p < 0.05$.
