# Supplementary material for: Diet-Nutrition Information Seeking, Source Trustworthiness, and Eating Behavior Changes: An International Web-Based Survey
Source: Nutrients. 2023 Oct 25;15(21):4515. doi: 10.3390/nu15214515 (PMC10649819; doi:10.3390/nu15214515)
Supplement: Supplementary file 1 [file nutrients-15-04515-s001.zip › nutrients-2647282-supplementary/Supplementary Table S5_Ruani Reiss Kalea.pdf]

nutrients MDPI

Table S5. Comparison of dietary change frequencies per each of six subpar information sources relative to the number of books owned.

Source Reliance for Making Dietary Changes		Number of Physical and Digital Books Owned					Total Responses *n*
		0-9	10-99	100-499	500-999	1000 or more	
		%					
**Social media**	Never	**6.4**	**32.4**	**40.3**	**13.4**	**7.6**	1243
	Rarely	**9.2**	**37.6**	**36.8**	**10.3**	**6.1**	620
	Sometimes	**10.3**	**47.9**	**30.8**	**7.7**	**3.3**	428
	Often	**26.4**	**34.1**	33.0	**5.5**	**1.1**	91
	All the time	20.7	58.6	13.8	0.0	6.9	29
**Influencers followed on social media**	Never	**7.0**	**32.3**	**40.1**	**13.2**	**7.5**	1288
	Rarely	**8.3**	**40.3**	**36.2**	**8.9**	**6.4**	531
	Sometimes	**12.8**	**41.9**	32.8	**8.7**	**3.9**	461
	Often	**15.8**	**48.3**	**28.1**	7.0	**0.9**	114
	All the time	15.6	**53.1**	**21.9**	6.3	3.1	32
**Famous personalities, actors, or presenters**	Never	**7.6**	**34.9**	**38.4**	**12.3**	6.7	1707
	Rarely	**8.9**	**40.8**	**35.9**	9.1	5.4	449
	Sometimes	**13.5**	41.1	**33.8**	**7.3**	4.4	207
	Often	**26.8**	**51.2**	**14.6**	4.9	2.4	41
	All the time	35.0	45.0	**20.0**	0.0	0.0	20
**Diet or health books**	Never	**15.0**	38.1	33.5	9.2	4.2	260
	Rarely	**10.4**	**37.0**	**33.7**	12.3	6.6	422
	Sometimes	**7.0**	**34.4**	**39.4**	**12.2**	7.1	1120
	Often	**7.4**	**40.2**	**38.2**	**9.7**	4.4	497
	All the time	11.4	**48.3**	**27.2**	6.1	7.0	114
**Nutrition or health websites**	Never	**13.4**	**35.0**	**32.3**	**12.4**	7.0	186
	Rarely	**9.0**	**33.3**	**37.3**	**13.3**	7.1	421
	Sometimes	**6.7**	**36.1**	**40.1**	**10.8**	6.3	1199
	Often	**10.6**	**40.3**	**33.1**	**11.3**	4.7	489
	All the time	**15.9**	**49.2**	**25.4**	**4.0**	5.6	126
**Google or Internet searches**	Never	**7.4**	**32.6**	**41.7**	12.0	6.3	460
	Rarely	**7.5**	**34.8**	**34.8**	**14.1**	**8.9**	655
	Sometimes	**7.5**	**39.4**	**38.0**	**9.8**	**5.3**	947
	Often	**14.1**	39.1	**34.4**	**8.7**	**3.6**	276
	All the time	**25.0**	**47.4**	**18.4**	**5.3**	4.0	76

* Bolded values represent $p < 0.05$.
